# Counting soil microbial communities: the impact of qPCR platform and mastermix on accuracy and precision

**DOI:** 10.1093/femsec/fiaf073

**Published:** 2025-07-24

**Authors:** Aoife M Duff, Madeline Giles, Syaliny Ganasamurthy, Antonia Santos, Sergio E Morales, Fiona Brennan

**Affiliations:** Department of Environment, Soils and Land-Use, Teagasc, Johnstown Castle, Wexford Y35 TC97, Ireland; James Hutton Institute, Dundee DD2 5DA, Scotland; Department of Microbiology and Immunology, University of Otago, PO Box 56, Dunedin 9054, New Zealand; Teagasc, Food Research Centre, Ashtown, Dublin D15 KN3K, Ireland; Department of Microbiology and Immunology, University of Otago, PO Box 56, Dunedin 9054, New Zealand; MPG Ranch, LLC, 19400 Lower Woodchuck Rd, Florence MT 59833, United States; Department of Environment, Soils and Land-Use, Teagasc, Johnstown Castle, Wexford Y35 TC97, Ireland

**Keywords:** environment, optimization, quantitative PCR, taq polymerase, validation

## Abstract

Quantitative polymerase chain reaction (qPCR) is widely used in soil microbial ecology to quantify microbial communities, but its accuracy can be compromised by coextracted inhibitors. Furthermore, large-scale international studies involving multiple laboratories or meta-analyses studies can introduce variation in qPCR results when data generated from different sources are compared. This study evaluated the performance of four commercial mastermixes across different soil types, a mock community, and a positive template control against three targets on three widely used platforms. Sensitivity to inhibitors was tested, with one mastermix affected, although this was mitigated by adding 1 mg/ml bovine serum albumin. Amplification success varied by mastermix, platform, gene, and sample matrix. Most mastermix–platform combinations showed low accuracy emphasizing the need for careful pairing. Precision was primarily influenced by gene target, followed by platform, sample matrix, and mastermix, and was reduced at lower template concentrations. Only 64.67% of intraassay (within an assay) measurements meet accepted thresholds. Interassay (between platforms) quantification was unreliable due to significant variability, which increased the risk of inaccurate data interpretation. The study highlights the necessity of considering inter- and intraassay variation, assay accuracy, and inhibitors that may impact sample amplification when utilizing qPCR for quantification of microbial communities in environmental samples.

## Teaser

qPCR is used to measure soil microorganisms but can be affected by inhibitors, change in mastermix, and platform. This study measured precision and accuracy across various platforms, mastermixes and gene targets.

## Introduction

Quantitative polymerase chain reaction (qPCR) is a molecular technique used ubiquitously for the absolute or relative quantification of targeted genetic sequences. Its high specificity, sensitivity, reproducibility, and wide detection range combined with its rapidity and high throughput nature has made it the gold standard technique for many applications across clinical, forensic, biotechnological, food science, and microbiological disciplines. The widespread use of qPCR to quantify taxonomic and functional genetic markers within environmental samples has revolutionized our understanding of the abundance, diversity, and activity of microbial communities in the environment, and their role in ecosystem functioning.

However, qPCR quantification of microbial communities within environmental samples can often pose distinct challenges. Environmental microbial communities, particularly those within soils, are often highly complex, resulting in divergent sequence targets, and requiring a delicate balance to be reached between specificity and coverage in the development of qPCR primers and probes (Smith and Osborn 2009). Environmental samples are also frequently characterized by coextraction of inhibitors, such as humic acids, the presence of which can interfere with downstream quantification by binding to nucleic acids, quenching the fluorescence signal, reducing the activity, or degrading the DNA polymerase (the enzyme responsible for the synthesis of the DNA molecule). This can result in inaccurate quantification, and the effect can be primer, target sequence, and Taq polymerase specific (Ijzerman et al. 1997, Daniell et al. 2012, Sidstedt et al. 2015, 2017). Further, endogenous controls that are frequently used to correct for extraction or amplification efficiency, as part of good qPCR practice within other sample types, do not exist for environmental samples (Chapman and Waldenström 2015). In the absence of better alternatives, the 16S rRNA gene is often used for normalization purposes in microbial ecology, however it does not meet the criteria for an endogenous control as it is not present or expressed at a constant level across treatments (Chapman and Waldenström 2015). The number of 16S rRNA copy numbers within the genome also varies widely across the microbial species so the response is a function of the makeup and successional stage of the community (Nemergut et al. 2016, Samad et al. 2017, Waters et al. 2018). For soils, another challenge is the lack of mock communities that represent the complexity of soil microbial communities.

The adoption of qPCR as a key tool for gene quantification has fostered the development of an array of commercial reagents and detection instruments, and an assortment of these have been used in microbial ecology studies. As efforts move towards an enhanced global understanding of the distribution and functioning of microbial communities, with an associated increase in large-scale international studies involving multiple laboratories or meta-analyses studies combining multiple independently generated datasets, it is crucial to understand whether data produced across different laboratories is comparable, and what the sources of intra (within) and inter (between) laboratory variability are. Optimizing qPCR assays can be time consuming and costly for many laboratories, therefore, many focus on developing new assays with a single mastermix without considering the specific target gene or level of sample inhibition (Fu et al. 2019, Barrat et al. 2021, Bahram et al. 2022, Zhou et al. 2022). As such, a key question is whether the differences in mastermix efficiencies and sensitivity to inhibitors may affect the ability to compare data across labs and between genes. While qPCR is ubiquitously used for quantification of taxonomic and functional communities across a range of soil types (Fu et al. 2019, Barrat et al. 2021, Bahram et al. 2022, Zhou et al. 2022, Frey et al. 2023), the accuracy (how close a measured value is to the true value) and precision (repeatability of measurements) of qPCR quantification across soils using a range of mastermixes, gene targets, and platforms has not previously been assessed to our knowledge. However, accuracy and precision assessments of other environmental samples, such as airborne microbial communities, revealed that qPCR assays underestimated true microbial aerosol concentration by a factor of 10–24, while coefficient of variation (%CV) ranged from 28% to 79% for three different test organisms (Hospodsky et al. 2010). This type of data is very helpful in that it will tell us how robust the assay is and whether more statistical rigour may be needed to improve accuracy and precision at the planning stage of an experiment. Further, the vast majority of environmental studies fail to report the essential and recommended MIQE (minimum information for publication of quantitative real-time PCR experiments) requirements (Bustin et al. 2009) for parameters associated with identifying intra- and interlaboratory variability, and inhibition. In this study, we evaluated the performance of four commercial qPCR mastermixes on three different detection platforms. We assessed their sensitivity to inhibitors, their capacity to accurately quantify genetic targets, and their precision by which they quantified environmental microbial communities by qPCR across a range of soil types and qPCR platforms.

## Materials and methods

### Soil descriptions and experimental design

Soils for this study were selected, across four sites in the southeast and midlands of Ireland, to encompass a range of edaphic properties (Table S1). This included a gradient of soil textural class and incorporated an organo-mineral, a clay, a loam, and a sandy loam soil. At each site a composite sample (comprising 15 soil cores, 10 cm depth) was collected between the months of April and May 2019. After homogenization in the field, a subsample was taken for molecular analysis. This was kept on ice following sampling and immediately frozen at −80°C on return to the laboratory. The remainder of the composite sample was air dried and sieved to 2 mm for physicochemical analysis. Further details on physicochemical analysis can be found in supplementary experimental procedures and Table S1.

Two main qPCR experiments were conducted. The first tested the relative sensitivity of four different mastermixes (Table 1) to naturally occurring inhibitors in the DNA extracts of each of the different soil types. This was conducted on a single qPCR platform (Biorad cfx384) in Teagasc Johnstown Castle Research Centre, Wexford, Ireland.

**Table 1.** Details of the mastermixes used in this study.

Mastermix name	Code	Company	Catalogue number
PowerUp™ SYBR™ Green Master Mix (PU)	PU	ABI	A25742
Takyon Low ROX SYBR 2X MasterMix blue dTTP–750 rxn (Tak)	Tak	Eurogentec	UF-LSMT-B0701
SsoAdvanced™ Universal Inhibitor-Tolerant SYBR® Green Supermix (SG)	SG	Biorad	1725016
Lightcycler 480 SYBR Green I Master (LC)	LC	Roche	04707516001

**Figure 1.**
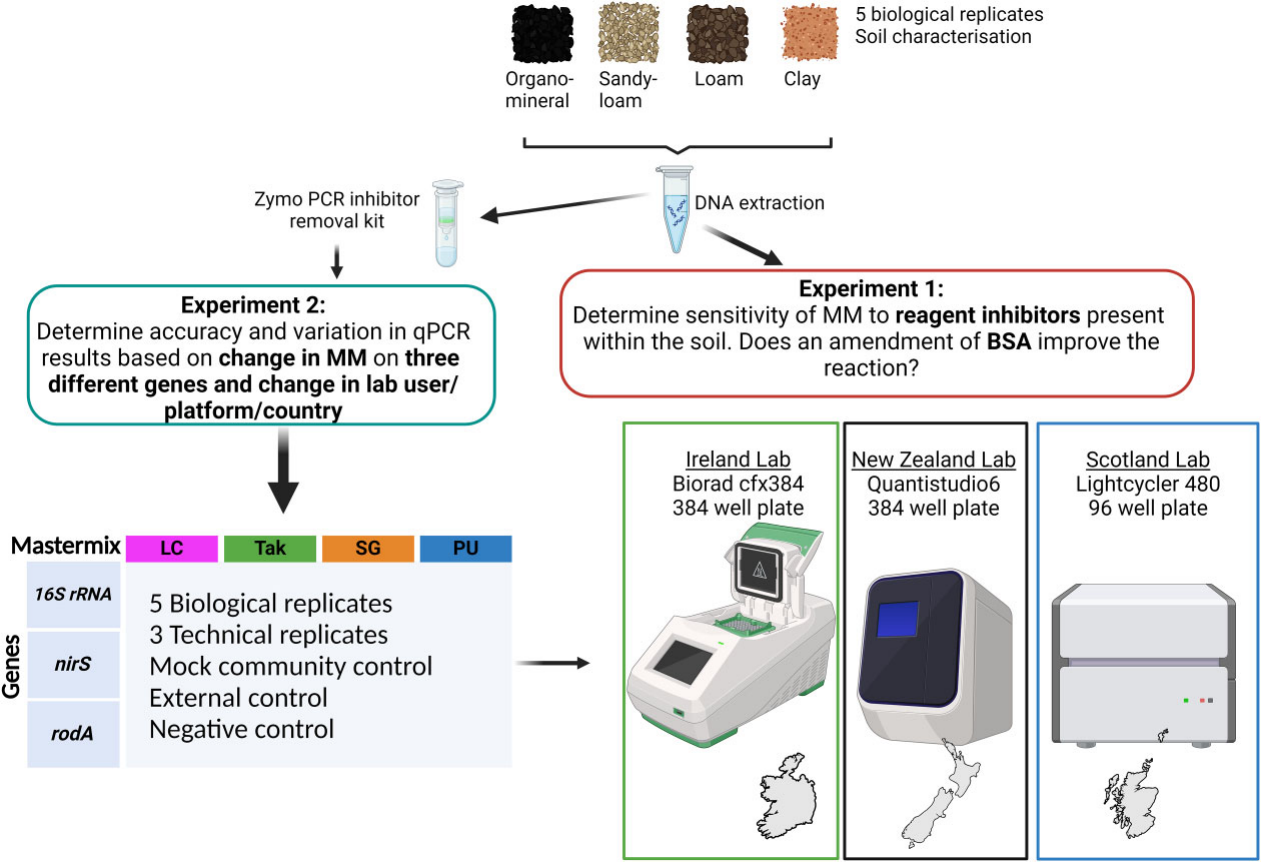
Flow diagram of the experimental design and setup four boxes indicate sample types taken organo-mineral, sandy loam, loam, and clay. The design is divided into two experiments: experiment one aims to determine the sensitivity of mastermixes to naturally occurring inhibitors within the soil; experiment 2 determines the accuracy and precision of quantifying three gene targets across mastermixes and platforms/users.

The second qPCR experiment aimed to test the capacity of qPCR assays to accurately and precisely quantify genetic targets across mastermixes and platforms. This was conducted in three laboratories Teagasc Johnstown Castle Research Centre, Wexford, Ireland; James Hutton Institute, Dundee, Scotland, UK, and the University of Otago, Dunedin, New Zealand, with each laboratory conducting the same assays on a different qPCR platform. Samples, standards, and controls were prepared at the Irish laboratory and aliquots were shipped on dry ice or using an ultra-freeze plus Bio-Bottle (Bio-Bottle Ltd., New Zealand) to laboratories in Scotland and New Zealand, respectively, where assays were run by local researchers on a Roche Lightcycler 480 and an ABI Viia7, respectively. Each Institute quantified three different genes using qPCR: a taxonomic gene, 16S rRNA; an environmental functional gene, *nirS*; and a housekeeping gene found in *Escherichia coli, rodA* (Table S2). The abundance of these genes were quantified in DNA extracted from the four soil types using the four mastermixes. Further, genes were quantified in a mock community (20 Strain Even Mix Genomic Material; ATCC, Ireland) and EC (detailed below), both containing a known abundance of the target genes. A schematic of the experimental design is depicted in Fig. 1.

### DNA extraction and inhibition testing

A total of 0.25 g of soil was extracted in replicates (*n* = 5) from each soil type using the Qiagen Power Soil Kit (Qiagen, Ireland) according to manufacturer’s instructions, but using a FastPrep-24 (MP Biomedicals, Ireland) to perform the bead beating step at 5 m/s for 15 s. DNA was eluted in 100 µl elution buffer. Following extraction, the relative sensitivity of four different mastermixes to inhibitors in the DNA extracts was determined via a qPCR inhibition test. Samples were spiked with an artificial plasmid (pGEM-T, Promega, Ireland) that was quantified using T7F and M13R primers targeting the plasmid promoter region. The plasmid was spiked into all samples and assays were run using the four different mastermixes. Assays were run with and without the inclusion of 1 mg/ml (0.5 µl per reaction) bovine serum albumin (BSA) (Invitrogen, Thermofisher, Ireland), added to reduce the effects of inhibition. Inhibition was assessed as described in the data and statistical analysis section. Regardless of the levels of inhibition detected within soil extracts post extraction, all samples were subject to clean up using the PCR inhibitor removal kit (Zymo Research, UK) according to the manufacturer’s instructions prior to experiment 2. Cleaned up DNA was quantified using the Qubit fluorometer dsDNA BR (Broad-Range) Assay 3.0 (Thermo Scientific, Ireland) and normalized to 1 ng/µl.

### qPCR

DNA standard curves were constructed from the target gene (Table S2) according to Duff et al. (2022). Briefly, a one-in-ten dilution series of appropriate standard was used over a six point dynamic range from 10^7^ to 10^2^ gene copies/μl. Target genes (16S rRNA, *rodA*, and *nirS*) were amplified (technical replicates *n*=3; biological replicates *n*=5) from four different soil types, a mock community control (MC; 20 Strain Even Mix Genomic Material; ATCC) and a pure culture external control (EC). *Pseudomonas aeruginosa* genomic DNA (DSM50071; DSMZ, Germany) was the EC used for 16S rRNA and *nirS* while *E. coli* K-12 strain (ATCC 47076; ATCC) genomic DNA was used as an EC for the *rodA* assay. Each 10 µl qPCR reaction mixture contained 5 µl of the appropriate mastermix (Table 1), a final concentration of 0.2 µM (*rodA* and 16S rRNA) or 1 µM of each primer (*nirS*; Table S2) for the relevant primer set, and 2 µl template DNA (1 ng/µl). Triplicate no-template controls were included. The specificity of the amplicons was confirmed by melt curve analysis at the end of each qPCR experimental run. The slope, y-intercept, and *r*^2^ values of standard curves are reported in Table S3.

### Data and statistical analysis

Sample inhibition was assessed by quantifying the artificial plasmid spike (as described above). Inhibition, as assessed by ΔCt, was calculated as the difference in Ct (quantification cycle at which the sample reaction curve intersects the threshold line) values between the sample (plasmid and sample) and the control (plasmid and water). ΔCt, has a direct impact on quantification, and gives an indication of how inhibition or other sample related factors are impacting the accuracy of the quantification. Where there is no impact of the sample on quantification of the target gene the ΔCt would be zero. The Ct value was calculated using single threshold mode on the CFX Maestro software (Bio-Rad Laboratories, USA). ΔCt was then expressed as a percentage via the calculation of an expected recovery (ER) value calculated using the equation ER = (2^ΔCt^)^−1^ × 100%. For ER the closer the value is to 100% the less likely inhibitors or other sample related factors are impacting quantification. Statistical significance between the Ct values of the control and sample was assessed using the Mann–Whitney U test (stats package in R 4.3.2; Bauer 1972). In addition to a direct effect on the Ct value, inhibition can also impact on the shape of the amplification curves. To assess this, we calculated the amplification efficiency (E) value, a measurement of the relative shape of the amplification curve for each sample compared to the control, using the qPCR data as described in King et al. (2009). To calculate E, the Hill slope (the steepness of the amplification curve) was calculated using the equation ‘sigmoidal dose response curve’ on the qPCR amplification plots (raw fluorescence data) using the software GraphPad Prism version 9.1 (GraphPad Software, USA; Motulsky et al. 2004). E was thereafter determined for each sample reaction by expressing the Hill slope of each sample (plasmid and sample) as a percentage of the Hill slope of the control reaction (plasmid and water).

To determine the proportion of the assays undertaken that were amplified, the percentage of total reactions quantified was measured by counting the number of reactions that were quantified above zero divided by the total number of reactions processed multiply by 100%. This was split up into percentage measures per gene (*n* = 792), mastermix (*n* = 594), platform (*n* = 792), and sample matrix (*n* = 540 for soils; *n* = 180 for controls).

To assess the accuracy of the three different assays across mastermix, soil type, and platform, gene abundances of the MC and EC were quantified and compared to their theoretical number, percentage error (%error) was calculated using this formula:


\begin{eqnarray*}\% \textit{error} = \ \frac{{\textrm{Actual}\ \textrm{gene}\ \textrm{abundance} - \textrm{Theoretical}\ \textrm{gene}\ \textrm{abundance}}}{{\textrm{Theoretical}\ \textrm{gene}\ \textrm{abundance}}}\ X\ 100\%. \end{eqnarray*}

The higher the %error the less accurate the quantification was.

The precision of the assay was calculated as the % coefficient of variation (%CV; standard deviation/mean population), and was calculated from the gene copy number of the MC and EC technical replicates. The lower the %CV the more precise the assay. Intraassay variation was assessed by calculating the %CV amongst the technical replicates within a platform and mastermix for a given gene target; Interassay variation was assessed by calculating the %CV amongst technical replicates across platforms within a mastermix and gene target. We adopted the maximum %CV cut-off proposed by Pfaffl (2004), of being below 20% for intraassay variation and below 30% for interassay variation, as being indicative of acceptable variation for qPCR assays.

All data was tested for normality using the Shapiro–Wilk test. Due to the skewed data distribution of gene abundances, %CV, and %error across mastermix, soil type, and platform, a Kruskal–Wallis test was used followed by Dunn’s test, to understand pairwise comparisons using R 4.2.1 and the ‘stats’ package. The chi-squared statistic obtained from the Kruskal–Wallis test quantifies the discrepancy or difference in variances among the groups being compared. Higher values of the chi-squared statistic indicate larger discrepancies between the observed versus expected data. This in combination with the *P*-value was used to understand the most significant factors influencing the data.

## Results

### Sensitivity to inhibitors

From experiment 1 (sensitivity to inhibitors) Table 2 depicts the sensitivity of mastermixes to natural inhibitors in the soils as assessed by % ER and ΔCt values. Only one mastermix (SG) and two soil combinations (clay and loam) demonstrated significantly lower amplification of the sample compared to the control (*P* < .05).

**Table 2.** The mean percentage of expected recovery (%ER) and difference in cycle threshold (ΔCt) values after amplification of the control (plasmid and water) and the sample (plasmid and DNA sample) is presented. An asterisk (*) signifies a *P*-value < .05, indicating a significant difference between the sample and control Ct values, suggesting the mastermix is sensitive to inhibitors present in that soil type. For samples sensitive to inhibitors, 1 mg/ml BSA was added to the mastermix, and quantification was repeated.

Mastermix	ABI Power up SYBR (PU)		Light cycler SYBR green 1 Master (LC)		Eurogentec Takyon low rox SYBR dTTP (Tak)		Biorad SSo fast Universal SYBR (SG)			
BSA added	0 mg/ml BSA		0 mg/ml BSA		0 mg/ml BSA		0 mg/ml BSA		1 mg/ml BSA	
	ER (%)	ΔCt	ER (%)	ΔCt	ER (%)	ΔCt	ER (%)	ΔCt	ER (%)	ΔCt
Clay	84.6	0.436	95.3	0.075	102	−0.242	82.5	0.277*	86.3	0.215
Loam	95.1	0.278	106	−0.071	110	−0.139	74.8	0.434*	89.6	0.165
Organo-mineral	142	−0.312	107	−0.093	91.1	0.147	78.9	0.348	94.8	0.079
Sandyloam	109	−0.067	122	−0.28	106	−0.083	69.4	0.544	92.5	0.114

An asterisk (*) signifies a *P*-value < .05, indicating a significant difference between the sample and control Ct values, suggesting the mastermix is sensitive to inhibitors present in that soil type. For samples sensitive to inhibitors, 1 mg/ml BSA was added to the mastermix, and quantification was repeated.

One mg/ml BSA was added to the SG reaction to attempt to mitigate any sensitivity the mastermix had to inhibitors found in the soil and following the addition of BSA inhibition was effectively mitigated in all soil types.

The shape of the amplification curve was also assessed using %E. We found that inhibitors did affect %E (Fig. S1), however the shape did not influence %ER, which directly affects the gene copy number result.

### Evaluation of amplification success

Experiment 2 aimed to evaluate the precision and accuracy of the qPCR assays in quantifying different genetic targets across different sample matrices, mastermixes, and platforms.

**Figure 2.**
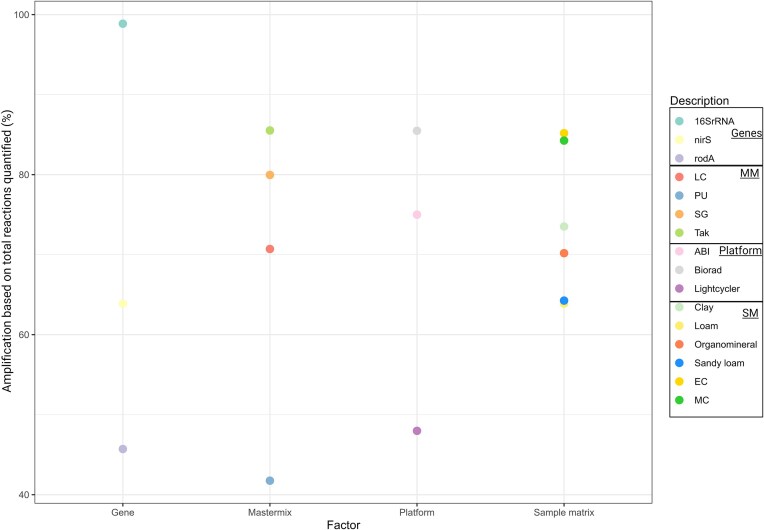
Scatter plot depicting % of total amplification success across genes, mastermixes, platforms, and sample matrices. Each variable is represented by distinct colours and categorized in the legend, abbreviations are as follows MM (mastermix); SM (sample matrix); EC (external control); and MC (mock community); (refer to Table 1 for details on Mastermix abbreviations used). Reactions were considered to have amplified if they had a greater amplification product than the negative control. This plot considers the presence but not the extent of amplification.

In some cases reactions were unsuccessful, with no amplification observed for given assays (above that of the negative controls) and the extent of these failed reactions differed between mastermixes, sample matrices, platforms, and genes (Fig. 2). A total of 594 reactions were run for each mastermix. Among these, the Tak mastermix amplified the highest number of reactions at 85%, compared to the PU mastermix, where amplification was only detected in 41% of reactions (Fig. 2). 792 reactions were run per gene; however, only 45% of *rodA* gene reactions successfully amplified compared to 98.8% for 16S rRNA reactions (Fig. 2). The Biorad platform had the greatest number of reactions amplified (*n* = 792 per platform), with amplification detected in 85% of reactions followed by 75% of reactions amplified by ABI and 47.9% by the Lightcycler platform. Finally, there was 540 reactions per soil type and 180 reactions for each control. Control reactions that successfully amplified ranged from 84% to 85% while soils ranged from 63% to 73% (Fig. 2).

### Accuracy across mastermix–platform combinations

**Figure 3.**
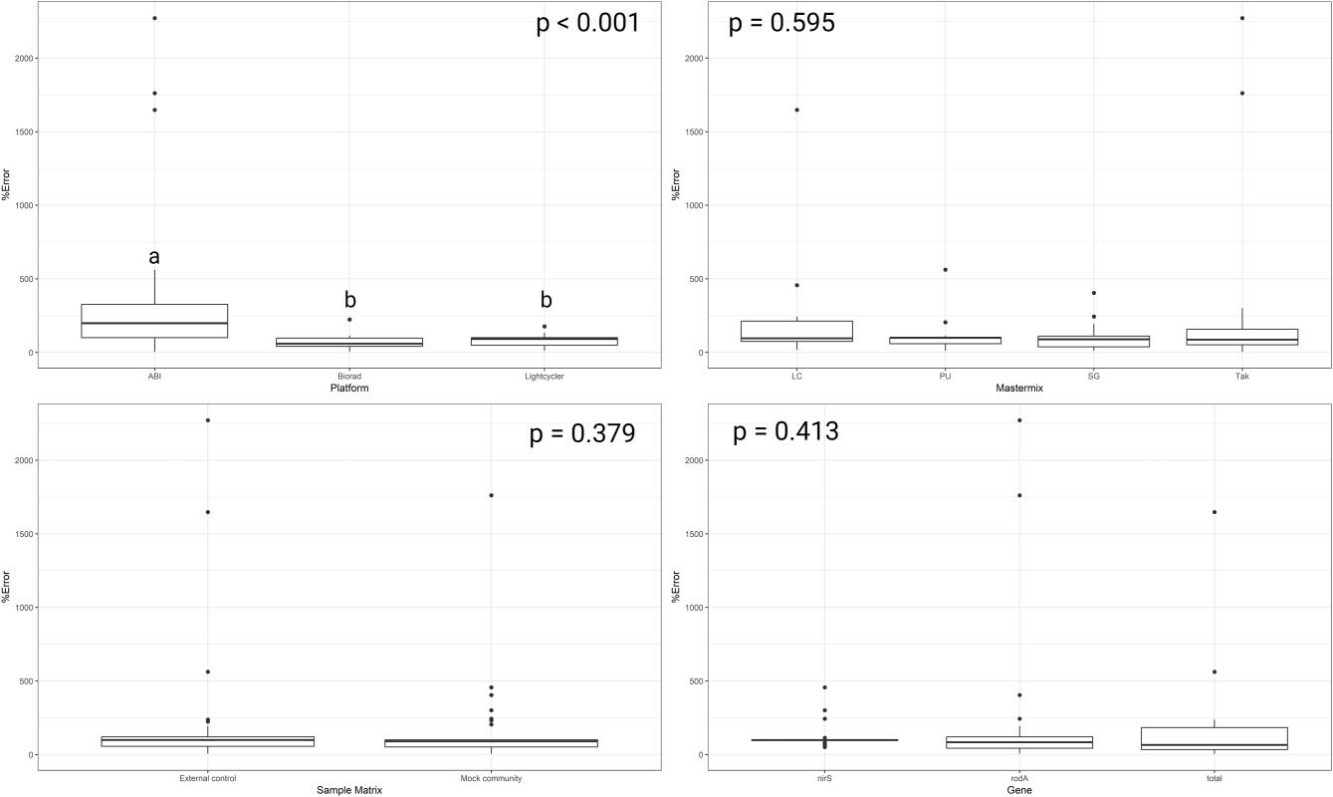
Determining Accuracy using %error of the mock community control (MC) and external control (EC) across platforms, mastermix, sample matrix, and genes. Mastermix abbreviations explained in Table 1. Box plot indicate the %Error (mean value with error bars ± standard deviation per platform (*n* = 24); mastermix (*n* = 18); sample matrix (*n* = 36); and gene (*n* = 24). *P*-values are indicated in the corner of each graph. Different small letters indicate data is significantly different from each other using the Dunn’s test.

When evaluating qPCR assay accuracy, the assessment was only conducted on the mock community and ECs, as these provided known theoretical values for each assay. Accuracy was low across platforms (*n* = 24) with %error for ABI ranging from 5% to 2271% (mean 405%; median 199%) while %error on the Biorad platform ranged from 6% to 223% (mean 68%; median 60%) and finally on the Lightcycler platform %error ranged from 10% to 176% (mean 78%; median 91%). ABI was significantly less accurate than both Biorad and Lightcycler platforms (*P* < .001; Fig. 3). There was no significant difference in accuracy across mastermix (*n* =18), however overall the % error was high for all mastermixes LC (mean 214%; median 96%); PU (mean 114%; median 100%); SG (mean 101%; median 89%), and Tak (mean 305%; median 86%). There was no significant difference in accuracy across genes either and %error continued to be high with 16S rRNA (mean 174%; median 65%), *nirS* (mean 122%; median 98%), and finally *rodA* (mean 254%; median 83%). Finally, sample matrix showed no significant difference in accuracy when comparing EC and MC controls (Fig. 3). The most accurate combinations included the ABI platform with Tak and SG mastermixes; the Biorad platform with SG, Tak, and PU mastermixes; and the LightCycler 480 platform with LC and SG mastermixes. These combinations produced %error values below 20% at least once. Additionally, SG was the only mastermix to achieve accurate quantification with %error below 20% on all three platforms.

### Sources of variation

We evaluated precision of the qPCR assays in quantifying genetic targets across different sample matrices, mastermixes, and platforms to identify source of intra- and interassay variation

### Intraassay variation

**Figure 4.**
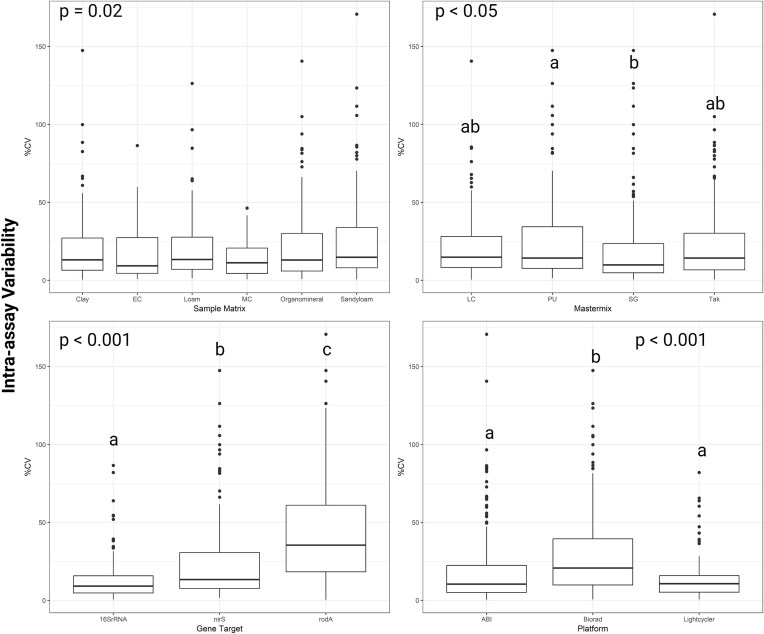
Determining precision using %CV that was calculated within an assay (intraassay). Variation was compared across platforms, mastermix, sample matrix, and genes. Mastermix abbreviations explained in Table 1. Box plot indicates the %CV intraassay (mean value with error bars ± standard deviation, total observations is 552, *n* varied per factor and is detailed in text. *P*-values are indicated in the corner of each graph. Different small letters indicate data is significantly different from each other using the Dunns’ test.

Intraassay was assessed by calculating the %CV amongst the technical replicates within a platform and mastermix for a given gene target. Any undetected quantifications were excluded from the analysis prior to calculating the %CV. Consequently, the sample size (*n*) varied for each variable. For the genes 16S rRNA assay (*n* = 262; mean 12.5%; median 9.1%) demonstrated significantly lower variability than *nirS* (*n* = 170; mean 24.1%; median 13.4%), while *rodA* performed poorly as an assay with the highest variability (*n* = 120; mean 43%; median 35.4%; Fig. 4). For the sample matrix variation was not significantly different across soil types or controls. For the mastermixes PU (*n* = 85; mean 27.8%; median 14.3%) variation was significantly higher than SG mastermix (*n* = 158; mean 19.6%; median 9.9%). Lastly, for the platforms, Biorad (*n* = 228; mean 30.2%; median 20.8%) demonstrated significantly higher variability than both Lightcycler (*n* = 126; mean 14.1%; median 10.7%) and ABI platforms (*n* = 198; mean 19.5%; median 10.4%; Fig. 4). In our analysis of the factors influencing %CV, the Kruskal–Wallis rank sum tests indicate that gene target had the most significant impact (chi-squared = 111.03, *P*-value < 2.2e-16), followed by platform type (chi-squared = 47.144, *P*-value = 5.791e-11), and mastermix choice (chi-squared = 10.904, *P*-value = 0.01226). Sample matrix did not significantly influence the data (chi-squared = 7.2328, *P*-value = 0.2039).

### Interassay variation

**Figure 5.**
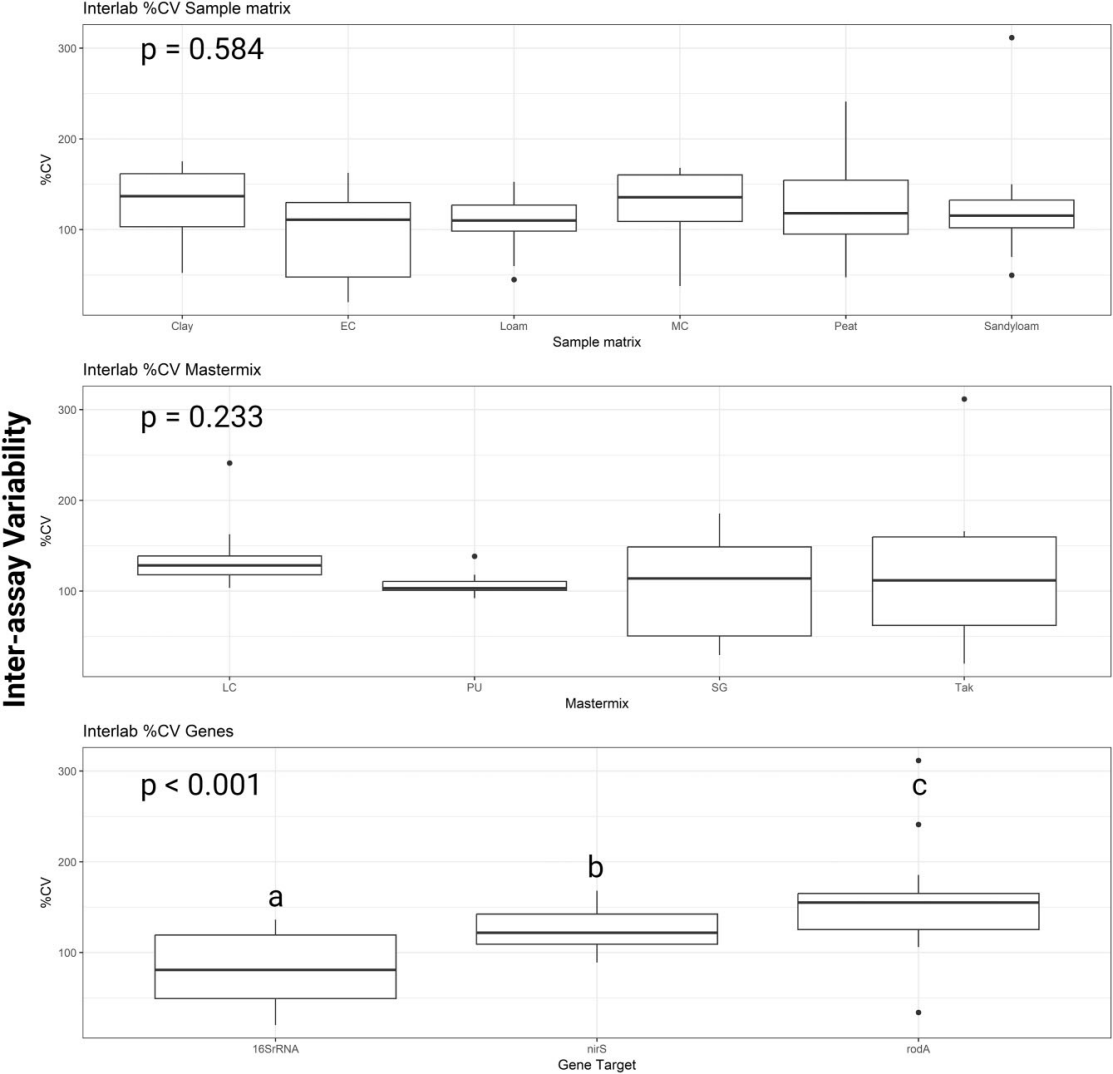
Determining precision using %CV that was calculated between assays or between platforms (interassay). Variation was compared across mastermix, sample matrix, and genes. Mastermix abbreviations explained in Table 1. Box plot indicates the %CV interassay (mean value with error bars ± standard deviation, total observations is 61 *n* varied per factor and is detailed in text. *P*-values are indicated in the corner of each graph. Different small letters indicate data is significantly different from each other using the Dunn’s test.

The variation observed between different platforms, known as interassay variation (*n* = 61; mean 117.9%; median 119%), was significantly higher compared to the intraassay variation (*n* = 552; mean 22.7%; median 13.2%; *P*-value < .001). Despite substantial variability across all factors, only the gene target exhibited a statistically significant difference in variability, with *rodA* (*n* = 18; mean 156%; median 155%) demonstrating the highest variability and the 16S rRNA gene assay (*n* = 24; mean 82.2%; median 81.1%) the lowest. However, even the gene with the least variability exceeded the 30% threshold considered viable when comparing quantitative data across platforms (Fig. 5). Ultimately, 64.67% of the intraassay %CV results were below the 20% threshold while only 3% of the interassay %CV results were below the 30% threshold.

### Quantifying soil microbial community as impacted by mastermix and platform

**Figure 6.**
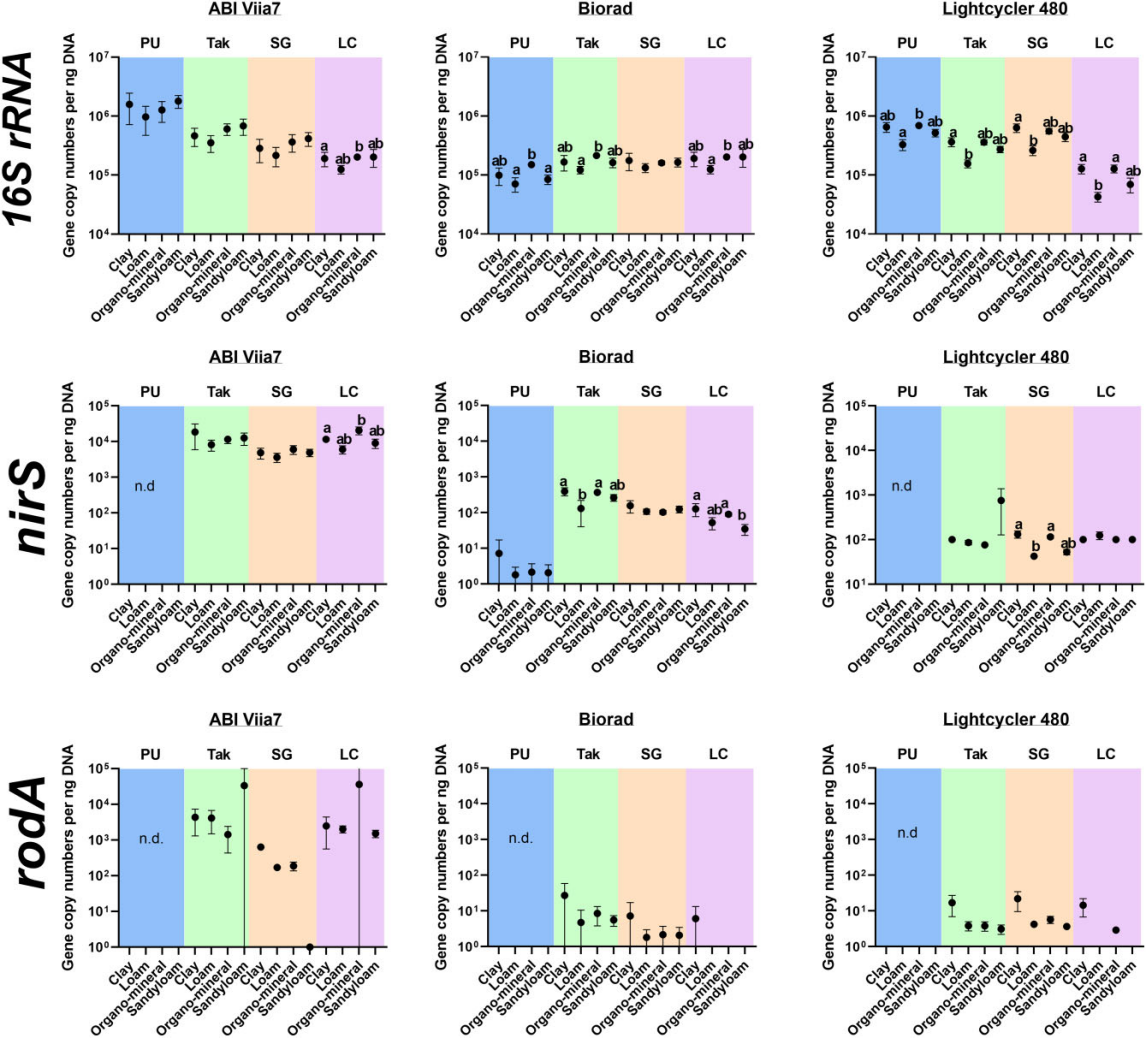
Demonstrating trends in gene abundances across soil types within a mastermix using Log gene copy numbers of the different four soil types for three different assay *16S rRNA* (a), *nirS* (b), and *rodA* (c). Results are grouped by platform mastermix abbreviations are explained in Table 1 PU (blue background), Tak (green background), SG (orange background), and LC (pink background). Soil type are shown on the *x*-axis Clay, loam, organo-mineral, and sandy loam soils. The black circles are the actual reading (gene copy number) mean value with error bars ± standard deviation (*n* = 5). The lower-case letters represent intraassay statistical differences between gene abundances (within a platform and mastermix) across soil types.

When analysing trends across soil types within a specific mastermix and platform (Fig. 6), the 16S rRNA assay exhibited the most significant differences, with 8 out of 12 platform and mastermix combinations showing significant variations between soil types. Notably, on the ABI platform, no significant differences were observed between soil types for the PU, Tak, and SG mastermixes. However, a significant difference was found when using the LC mastermix (*P* = .018). For the Biorad platform, the PU (*P* = .006), Tak (*P* = .004), and LC (*P* = .025) mastermixes demonstrated significant differences among soil types. Consistently, the conclusion was that the abundance of the 16S rRNA gene was significantly lower in loam soil compared to organo-mineral soil. However, no significant difference was observed for the SG mastermix. In contrast, on the Lightcycler 480 platform, all mastermixes showed significant differences between soil types. Specifically, the SG (*P* = .039), LC (*P* = .012), and Tak (*P* = .023) mastermixes led to the conclusion that clay soil had significantly higher 16S rRNA gene abundance compared to loam soil. On the other hand, the PU mastermix indicated that loam soil had significantly lower abundance compared to organo-mineral soil, aligning with the findings on the Biorad platform.

In analysing the trends across soil types for *nirS* gene abundance within the platform and mastermix combinations (Fig. 6), only 4 out of 12 combinations showed significant differences across soil type. On the ABI platform, the LC combination revealed that clay soil had significantly lower *nirS* gene abundance compared to loam soil (*P* = 0.002), while the PU combination did not detect any gene copies. The Tak and SG combinations showed no significant differences among soil types. For the Biorad platform, the PU and SG combinations did not exhibit significant differences in *nirS* gene copy numbers between soil types. However, the Tak combination indicated that both clay and organo-mineral soils had significantly higher *nirS* gene abundances than loam soil (*P* = .005). In contrast, the Biorad platform in combination with the LC mastermix revealed that both clay and loam soils had significantly higher *nirS* gene abundances compared to sandy loam soil (*P* = .004). In terms of the Lightcycler platform, the PU mastermix did not detect *nirS* gene copy numbers in any of the soils. The Tak and LC combinations showed no significant differences (Fig. 6). However, the SG combination indicated that clay and organo-mineral soils had significantly higher *nirS* gene abundances than loam soil (*P* = .003), which corresponds to the Biorad platform and Tak mastermix combination. In contrast, the *rodA* assay showed no significant differences across all platform and mastermix combinations.

## Discussion

### Sensitivity to inhibitors observed in SG mastermix only

The aim of the initial phase of this study was to determine whether commonly used commercial mastermixes were sensitive to inhibitors. Soil samples are well known to contain various inhibitors including humic acids, fulvic acids, calcium ions, and iron chloride (Bickley et al. 1996, Ijzerman et al. 1997, Kuffel et al. 2021). Even at low concentrations, humic acids are known to directly impact Taq polymerase activity and have been shown to affect polymerase sensitivity more significantly than assay factors, such as primer selection and target sequences (Ijzerman et al. 1997, Sidstedt et al. 2017). This study underscores the importance of testing for inhibitors, as the commercial mastermix SG exhibited significant sensitivity to soil inhibitors, which might have gone unnoticed otherwise. On the other hand PU, Tak, and LC were not sensitive to the inhibitors found in all four soil types. Similarly, Albers et al. (2013) demonstrated that six different commercial mastermixes tolerated varying humic acid concentrations ranging from 0.05 to 10 mg/l humic acid substances before exhibiting evidence of inhibition. Strategies for removal of inhibitors include additional extraction steps, such as chloroform extraction, treatment with activated carbon, PEG precipitation, and use of inhibitor removal kits (Schrader et al. 2012, Hunter et al. 2019, Sidstedt et al. 2020). Additionally, during the PCR stage a more general and widely applied method is to dilute the sample of the extracted DNA with the expectation that the PCR inhibitors will also be diluted. However, the dilution of the DNA can lead to decreased sensitivity. Other approaches of mitigating the impact of inhibitors on amplification is by adding thermostable taq polymerases, BSA, optimal magnesium ions or the T4 bacteriophage gene 32 product to the PCR reaction (Kreader 1996, Baar et al. 2011, Sidstedt et al. 2015, Kuffel et al. 2021). Since BSA is a cost-effective and easy strategy to reduce inhibitory effects, the second part of this experiment tested its capacity to alleviate mastermix sensitivity to inhibitors. BSA is a protein that binds strongly to organic molecules, including compounds such as phenols and humic acids thereby protecting the taq polymerase during the PCR (Kreader 1996). Our study found that the addition of 1 mg/ml BSA to the qPCR reaction for SG mastermix significantly improved amplification by 3.8% and 12.1% for the clay and loam soil, respectively (*P* < .05). Albers et al. (2013) demonstrated only two out of six mastermixes did not improve mastermix tolerance to inhibitors after BSA addition and suggested a possible reason for this was the formation of hydrogen bonds with phenolic compounds found in humic substances. However, even with numerous strategies to combat inhibition many studies on soil report qPCR results without evaluating inhibition, potentially resulting in inaccurate quantification (Fu et al. 2019, Barrat et al. 2021, Bahram et al. 2022, Zhou et al. 2022).

### Accuracy was low across most mastermix–platform combinations

We further evaluated whether the accuracy in gene quantification varied across assays (three genes) based on change in mastermix in platform. Overall, accuracy was very low, which is of concern as many studies do not report validation checks confirming accurate quantification (Fu et al. 2019, Zhou et al. 2022, Frey et al. 2023). When comparing across platforms (platform, lab user, and country were considered confounding factors and referred to as platform variation throughout the study). Mean % error was 405%, 67%, and 78% for ABI, Biorad, and Lightcycler platforms with the ABI platform having a significantly higher %error than the other two platforms. Similarly, other studies also found that comparing across platforms often yielded significantly different results and inaccurate data (Buzard et al. 2012, Ebentier et al. 2013). Percentage error was also high for genes, and mastermix and sample matrix but there was no significant difference within each factor. The pairing of the mastermixes with the platform was found to be important. The most accurate combinations included the ABI platform with Tak and SG mastermixes; the Biorad platform with SG, Tak, and PU mastermixes; and the LightCycler 480 platform with LC and SG mastermixes. These combinations produced %Error values below 20% at least once. It is important to consider both accuracy and precision in the context of the proportion of failed assays, as in some cases the precision and accuracy may be artificially inflated by the number of failed assays, which were not considered as part of the calculations. For e.g. the PU mastermix had the lowest number of reactions, where amplification occurred but where amplification did occur the quantification was considered accurate. In terms of amplification success, the Lightcycler platform was the poorest performer with only 48% of reactions amplified but similarly to the other mastermixes it also had poor accuracy when quantifying the *nirS* gene.

Standard curve efficiency is reported as a measure of how well an assay is performing, however, a good standard curve efficiency (between 90% and 110%) does not guarantee accurate results. In many cases, the environmental sample efficiency can differ significantly from the standard curve efficiency yielding inaccurate results (Pfaffl 2001, Ruijter et al. 2009, Towe et al. 2010, Brankatschk et al. 2012). Our study-demonstrated mastermixes SG and LC had 93.7% and 95.9% efficiencies, respectively and both assays underestimated *nirS* gene abundances on the biorad platform by 2 orders of magnitude (Fig. S2, Table S3). Indeed, often the results did not follow the same trend as the efficiency i.e. low efficiencies overestimated rather than underestimated the absolute value for e.g. frequently with the PU mastermix. These discrepancies using the standard curve method were investigated in other studies and solutions to deal with them were proposed, such as the one-point calibration method, adjusting of fluorescence baselines, and the Pfaffl method (Pfaffl 2001, Ruijter et al. 2009, Brankatschk et al. 2012). However, the standard curve method remains the gold standard in environmental studies. A standard set of conditions was used across mastermixes and platforms to minimize variables, though assays are typically optimized for efficiency. Thus, this study focuses on standard rather than optimized conditions so further improvements in efficiency could be expected.

### Gene target is the greatest source of variation in both intraassay and interassay variation

Precision is also essential for assay validity, as poor precision can obscure true differences between treatments, leading to incorrect conclusions. In this study, the precision of each assay (16S rRNA, *nirS*, and *rodA*) was determined using four different mastermixes and the instrument variation of the assays. Intraassay variation in %CV between 0.46% and 86.6% were observed for the 16S rRNA assay with the use of four different mastermixes and three platforms; 1.5% to 147.4% for *nirS* and 0.4% to 170.7% for *rodA*. In a study quantifying 16S rRNA in coastal and river sediments *E. coli, Aspergillus fumigatus*, and *Bacillus atrophaeus* on air filters, %CVs ranged from 28% to 79% when targeting concentrations of bacteria at 10^3^ and 10^4^ gene copy numbers (Hospodsky et al. 2010). From Hospodsky et al. (2010), Smith et al. (2006), and this study, it is clear that the %CV does increase when targeting template at lower concentrations as 16S rRNA had significantly lower %CV overall and targeted higher gene copy concentrations compared to the *rodA* and *nirS* gene targets (*P* < .001). However, the low template may not be the only factor driving an increase in %CV as *nirS* gene concentration was in a similar range to *rodA* and had a higher %CV in some cases. Our study highlights the numerous sources of variation in qPCR results, identifying gene target as the greatest source of variation, followed by platform, sample matrix, and mastermix. The degenerate nature of the *nirS* primer could be a factor as studies have shown that degenerate primers can significantly underestimate genes with primer sets showing up to a 10 000-fold misestimation (Bru et al. 2008, Gaby and Buckley 2017). Another study reported intraassay variation of 0.1%–3.3%, when quantifying a range of genes in faecal samples, however, it was not clear if the %CV was calculated with Ct values or gene copy numbers (Ebentier et al. 2013). In this study, %CVs were calculated using gene copy numbers, this is an important distinction as %CVs of Ct values will always be lower and potentially misleading (Bustin et al. 2009). A key question is what should be considered an appropriate %CV cut off, as few studies provide recommendations. Pfaffl (2004) suggests 10%–20% and 15%–30% for interassay variation, while Broeders et al. (2014) recommended ≤25% and ≤30%, respectively. Our study emphasizes the importance of reporting %CV and/or confidence intervals, with gene copy numbers offering a more accurate measure of variability than Ct values.

Finally, we assessed quantification of three different gene targets in four different soil types using the same four mastermixes and three platforms as before. Overall, there was no consistent trend throughout all 12 platform and mastermix combinations, other than the combinations that showed no significant differences between soil types. However, the only consistent trend when soil types were significantly different was found when quantifying the 16S rRNA gene on the Biorad platform with PU, Tak, and LC mastermix demonstrating the same conclusion that 16S rRNA gene copy numbers were significantly higher in organo-mineral soil compared to loam soil. The choice of mastermixes and platform significantly affected gene copy number across soil types. If we look at the combinations of platforms and mastermixes for the soil extracts that were most accurate for mock community controls, Tak and SG paired with the ABI, Lightcycler480 and Biorad demonstrated accuracy using the mock community for the 16S rRNA gene, the 16S rRNA gene was the most consistent as both SG and Tak on the Lightcycler480 platform consistently gave the same results. On the ABI platform, Tak performed well in the mock community but yielded significantly higher gene concentrations for each soil type than the Biorad platform, raising concerns about which combination (if either) provides accurate quantification. Such variability complicates measuring absolute gene abundances, especially without proper controls to validate the quantification. Similarly, Smith et al. (2006) found that even statistically comparable standard curves in a single qPCR assay led to significantly different absolute gene copy values from the same template DNA. Consequently, we have demonstrated that absolute quantification of environmental samples must be approached with caution and a positive control of known gene copy number may be necessary to ensure absolute quantification of soil is possible using current qPCR methods. If a positive control is unavailable for an environmental assay, relative quantification could be an alternative, as suggested by Daniell et al. (2012). This method involves spiking an artificial plasmid into soil before the DNA extraction to serve as an internal positive control, correcting for DNA extraction efficiency and qPCR performance. However, it may increase variability, as spike recovery varied significantly across soil types, ranging from 2.61% to 13.66% (Daniell et al. 2012).

## Conclusion

We found that PCR inhibition was only observed in the SG taq but this was alleviated with the addition of BSA. Amplification success varied across mastermixes, platforms, genes, and sample matrices. Accuracy was found to be low in most mastermix–platform combinations, making the pairing of the mastermix–platform important for correct quantification. Precision was most strongly influenced by gene target type followed by change in platform, sample matrix, and finally the mastermix. %CV was impacted by the template concentration, with lower performance at lower template levels. We found that assays generated from different laboratories cannot be compared as the extent of variation (%CV over the acceptable threshold in the majority of samples) may obscure biological patterns, with potentially significant implications for meta-analysis studies. From the findings of this study, we would recommend that future qPCR studies: (i) Mitigate PCR inhibition by testing for inhibitors and using additives such as BSA where necessary. (ii) Carefully select and validate mastermix–platform combinations, as their interaction significantly affects accuracy and amplification success. (iii) Assess and report both inter- and intraassay variation, using %CV to quantify precision. We suggest %CV values are incorporated into experimental design to determine the level of technical replication needed to achieve acceptable variability. (iv) Include ECs such as a mock community or pure culture of known gene copy numbers to monitor assay performance and detect technical issues. (v) Avoid direct comparisons between assays from different laboratories unless protocols are standardized and cross-validated. Lastly, our results highlight the need for standardized methods within microbial ecology qPCR to ensure data accuracy, precision, and comparability, leading to high quality meaningful results.

## Supplementary Material

fiaf073_Supplemental_Files
