# Supplementary material for: Counting soil microbial communities: the impact of qPCR platform and mastermix on accuracy and precision
Source: FEMS Microbiol Ecol. 2025 Jul 24;101(8):fiaf073. doi: 10.1093/femsec/fiaf073 (PMC13223730; doi:10.1093/femsec/fiaf073)
Supplement: fiaf073_Supplemental_Files [file fiaf073_supplemental_files.zip › Supplementary Data.docx]

**Supplementary Experimental procedures**

**Physicochemical parameters**

Percentage water content of the soils was calculated immediately upon return to the lab. Approximately 20 g of soil was weighed and the exact weight was recorded. The samples were dried in an oven at 105°C overnight and thereafter placed in a desiccator until the sample was cool and ready to be re-weighed. The % Water Content (%WC) was calculated as follows %WC = (Weight of Water / Dry Weight of Soil) × 100.

Soil texture was classified using the soil particle size analysis pipette method (Dane and Topp et al., 2002). Briefly, 20 g of air-dry soil was weighed into 8L beakers and soil organic matter oxidation was performed. 150 ml 8% H_2_O_2_ was added to the soil and was heated to almost boiling point. 50-80 ml 8% H_2_O_2_ was added every so often until the frothing stopped. The final solution was reduced to approximately 100 ml before adding 3% ammonium hydroxide to decompose any remaining H_2_O_2_. 5% sodium hexametaphosphate (HMP) was added to the samples to facilitate flocculation of soil particles and the soil solution was shaken on an over-end shaker overnight. Samples were then poured and washed through a 2 mm and 0.05 mm sieve into a 1L graduated cylinder which was shaken and placed in a water bath at 22°C. A 22-ml aliquot was pipetted from the suspension, at depth of 7.5 cm from the surface. This subsample was reflective of the silt and clay content. 5 hours 43 minutes and 16 seconds later another 22 ml aliquot was taken in the same way this subsample reflected clay only. Both subsamples and sieves were left to dry at 105°C overnight. The soil fractions were calculated using the following calculations: %_𝑪𝒔𝒂𝒏𝒅=Csand(g) ∗ 100 Total soil fractions(g)

%_𝑭𝒔𝒂𝒏𝒅=Fsand(g) ∗ 100 Total soil fractions(g)

%_𝑺𝒊𝒍𝒕={[Silt&Clay(g)]−Clay(g)} ∗ 45.45∗ 100 Total soil fractions(g) %_𝑪𝒍𝒂𝒚=[Clay(g)−0.022] ∗ 45.45 ∗ 100 Total soil fractions(g)

Once calculated the USDA soil texture triangle was used to determine soil textural class. Gravimetric water content was determined by weighing the soil before and after drying at 105°C overnight.

For the remaining physico-chemical analysis, soil was dried at 40 °C for 3 days and sieved to 2 mm before analysis. Organic matter (OM) content was determined by combustion at 500 °C for 16 h. Soil pH was determined using a 2:1 ratio of deionised water to soil (Peyton et al., 2016). Samples were ball milled for 2 min at 23 Hz (180 - 1500 min^−1^) using a MM 200 Model Mixer Mill (Retsch, UK) before measuring total nitrogen (TN) and total carbon (TC) using the high-temperature combustion method by a LECO TruSpec CN analyser (Elementec, Ireland).

**Supplementary Tables:**

**Table S1: Sample location and Soil properties**

| **Soil** | **Date collected** | **Location** | | **Location Description** | **Water Content (%)** | **Organic Matter (%)** | **Total Carbon (%)** | **Organic Carbon (%)** | **Total Nitrogen (%)** | **pH** | **Textural class** |
| --- | --- | --- | --- | --- | --- | --- | --- | --- | --- | --- | --- |
| **Sandy Loam** | 03/04/2019 | N52°17'59.4" | W006°30'23.7" | Field 46 Dairy farm | 30.11 | 5.75 | 2.22 | 1.62 | 0.273 | 5.61 | Sandy loam |
| **Organo-mineral** | 05/05/2019 | N53°02'10.0" | W007°22'24.4" | Ross Bog Co. Laois | 49.37 | 17.03 | 10.2 | 8.57 | 0.608 | 6.06 | Sandy clay loam |
| **Loam** | 14/05/2019 | N52°17'35.3" | W006°30'03.3" | Cricket field | 28.64 | 7.21 | 3.02 | 2.29 | 0.297 | 5.69 | Loam |
| **Clay** | 14/05/2019 | N52°17'39.9" | W006°31'06.0" | Foals house | 33.77 | 8.26 | 3.72 | 2.87 | 0.335 | 6.61 | Clay |

**Table S2: Primer sets used in this study**

|  |  | ***Primers*** | |  |  |  |  |
| --- | --- | --- | --- | --- | --- | --- | --- |
| **Gene name** | **Function** | ***Forward*** | ***Reverse*** | **Conditions** | **Target length** | **Primer GC content (%)** | **Reference** |
| **Plasmid** | Artificial plasmid used for cloning purposes | T7F-TAA TAC GAC TCA CTA TAG GG (0.2 µM) | M13R-CAG GAA ACA GCT ATG AC (0.2 µM) | 95°C, 5min; 40×(95°C, 30s; 57°C 30s; 72°C, 30s, 85°C with plate read, 1s); Melt curve 65°C to 95°C, increment 0.5°C, 5 sec + plate read | 197 bp | 42% | Promega PGem-T vector systems protocol |
| **16S *rRNA* (bacteria)** | Taxonomic identifier | 341f- CCT ACG GGN GGC WGC AG (0.2 µM) | 785r- GAC TAC HVG GGT ATC TAA TCC (0.2 µM) | 95°C, 5 min; 40×(95°C, 40s; 55.6°C 1 min; 72°C, 30s, 80°C with plate read, 3s); Melt curve 65°C to 95°C, increment 0.5°C, 5 sec+ plate read | 444 bp | 61% | Klindworth et al., 2013 |
| ***nirS*** | nitrite reductase gene functional marker | cd3AF- GTS AAC GTS AAG GAR ACS GG (1 µM) | R3cd- GAS TTC GGR TGS GTC TTG A  (1 µM) | 95°C, 10min; 40×(95°C, 30s; 60°C 30s; 72°C, 20s, 79°C with plate read, 2s); Melt curve 65°C to 95°C, increment 0.5°C, 5 sec + plate read | 406 bp | 49% | Throback et al., 2004; Yergeau et al., 2004 |
| ***rodA*** | Peptidoglycan glycosyltransferase *Escherichia coli* specific housekeeping gene | rodA984F – GCA AAC CAC CTT TGG T (0.2 µM) | rodA984R- CTG TGG GTG TGG ATT GAC AT  (0.2 µM) | 95°C, 5 min; 40×(95°C, 10s; 66°C 20s; 72°C, 20s, 80°C with plate read, 2s); Melt curve 65°C to 95°C, increment 0.5°C, 5 sec + plate read | 120 bp | 49% | Chern et al., 2011 |

**Table S3: Assay performance indicators across mastermixes (ABI Power up SYBR (PU); Eurogentec Takyon low rox SYBR (Tak); SsoAdvanced™ Universal Inhibitor-Tolerant SYBR® Green Supermix (SG); Lightcycler SYBR green 1 Master (LC)) and detection platforms (Bio-Rad Cfx384; Lightcycler 480 and ABI Viia7) for *rodA*, *nirS* and 16S *rRNA* gene assays.**

|  | ***rodA*** | | | | ***nirS*** | | | | ***16S rRNA*** | | | |
| --- | --- | --- | --- | --- | --- | --- | --- | --- | --- | --- | --- | --- |
| ***Biorad cfx*** | *PU* | *Tak* | *SG* | *LC* | *PU* | *Tak* | *SG* | *LC* | *PU* | *Tak* | *SG* | *LC* |
| **Slope** | -5.896 | -3.68 | -3.354 | -3.209 | -4.985 | -3.614 | -3.482 | -3.425 | -4.631 | -3.727 | -3.575 | -4.616 |
| **Efficiency** | 47.8% | 86.9% | 98.7% | 104.9% | 58.7% | 89.1% | 93.7% | 95.9% | 64.4% | 85.5% | 90.4% | 64.7% |
| **Y intercept** | 61.453 | 38.953 | 35.549 | 37.355 | 46.733 | 35.708 | 34.004 | 35.650 | 44.017 | 38.090 | 35.611 | 45.626 |
| **r^2^** | 0.995 | 0.99 | 0.999 | 0.944 | 0.99 | 0.99 | 0.99 | 0.99 | 0.99 | 0.99 | 1 | 0.99 |
| **NTC** | 0 | 39.5 | 0 | 0 | 0 | 0 | 0 | 0 | 38.4 | 32.8 | 0 | 31.5 |
| ***Lightcycler*** | *PU* | *Tak* | *SG* | *LC* | *PU* | *Tak* | *SG* | *LC* | *PU* | *Tak* | *SG* | *LC* |
| **Slope** | -3.15 | -2.948 | -2.886 | -2.926 | 0 | -3.62 | -3.419 | -3.542 | -4.04 | -3.08 | -2.880 | -3.415 |
| **Efficiency** | 107.5% | 118.3% | 122.07% | 119.66% | 0 | 88.89% | 96.07% | 91.57% | 76.7% | 111.1% | 122.4% | 96.24% |
| **Y intercept** | 50.97 | 36.506 | 36.92 | 36.698 | 0 | 42.16 | 41.34 | 38.77 | 61.7 | 37.73 | 36.68 | 38.776 |
| **R^2^** | 0.99 | 0.98 | 0.984 | 0.997 | n/a | 0.98 | 0.984 | 0.995 | 0.947 | 0.98 | 0.987 | 0.997 |
| **NTC** | 0 | 35 | 0 | 0 | 0 | 0 | 0 | 0 | 0 | 0 | 32.2 | 0 |
| ***ABIViia7*** | *PU* | *Tak* | *SG* | *LC* | *PU* | *Tak* | *SG* | *LC* | *PU* | *Tak* | *SG* | *LC* |
| **Slope** | 0 | -4.14 | -4.42 | -4.82 | -6.68 | -4.79 | -4.4 | -5.73 | -4.27 | -4.06 | - 4.13 | -3.81 |
| **Efficiency** | 0 | 74.45% | 68.29% | 61.3% | 41.13% | 61.69% | 68.72% | 49.44% | 71.55% | 76.2% | 74.71% | 83.1% |
| **Y intercept** | 0 | 48.57 | 44.13 | 47.16 | 80.55 | 50.84 | 44.54 | 55.36 | 52.2 | 41.7 | 41.01 | 48.3 |
| **R^2^** | 0 | 0.917 | 0.952 | 0.917 | 0.555 | 0.989 | 0.987 | 0.555 | 0.827 | 0.955 | 0.99 | 0.754 |
| **NTC** | 0 | 33.3 | 0 | 0 | 0 | 0 | 0 | 0 | 0 | 31.5 | 32.5 | 0 |
