# Supplementary figures and images for: Counting soil microbial communities: the impact of qPCR platform and mastermix on accuracy and precision
Source: FEMS Microbiol Ecol. 2025 Jul 24;101(8):fiaf073. doi: 10.1093/femsec/fiaf073 (PMC13223730; doi:10.1093/femsec/fiaf073)

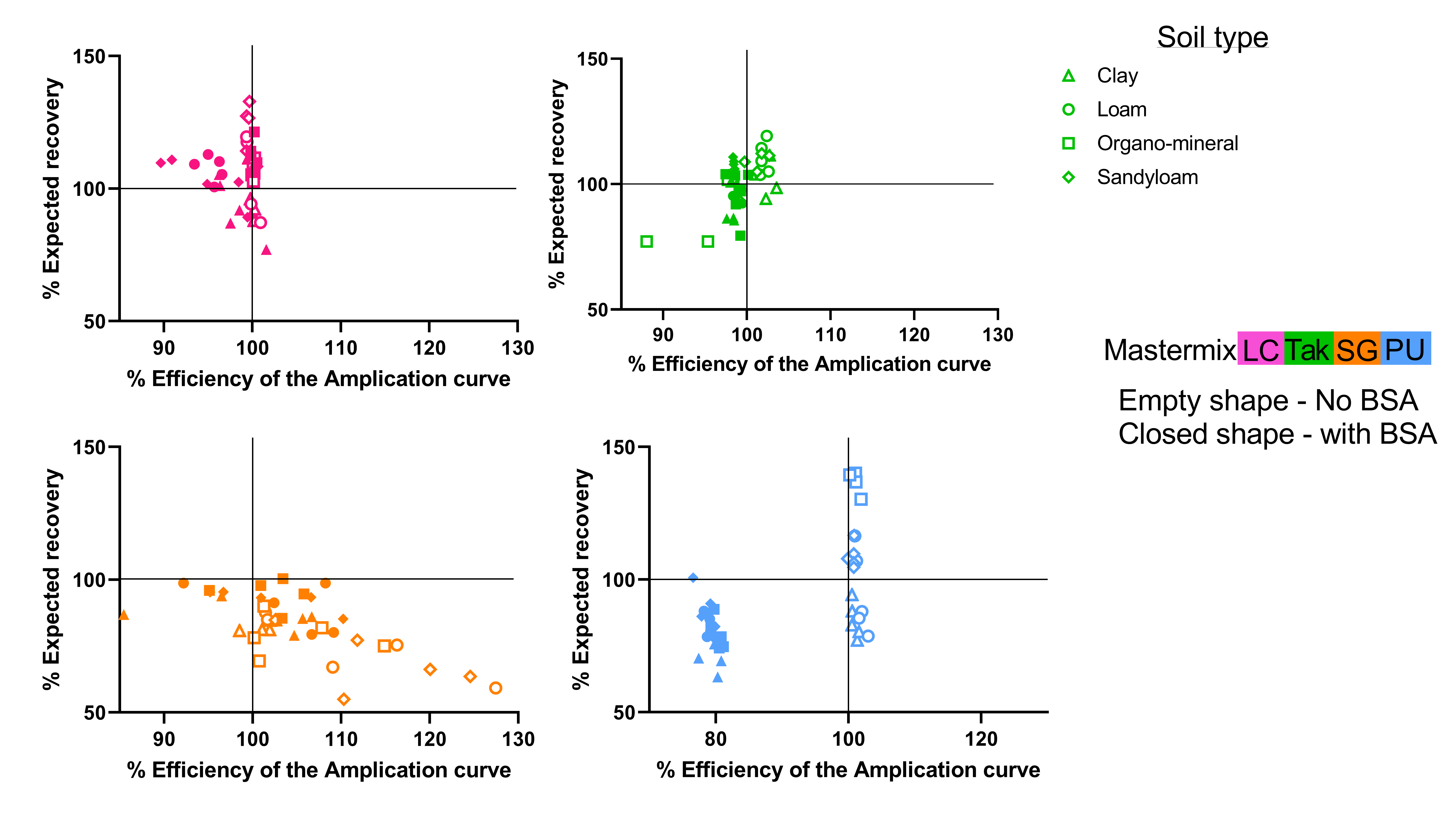

Supplement: fiaf073_Supplemental_Files [file fiaf073_supplemental_files.zip › Fig. S1 Supplementary Data.png]

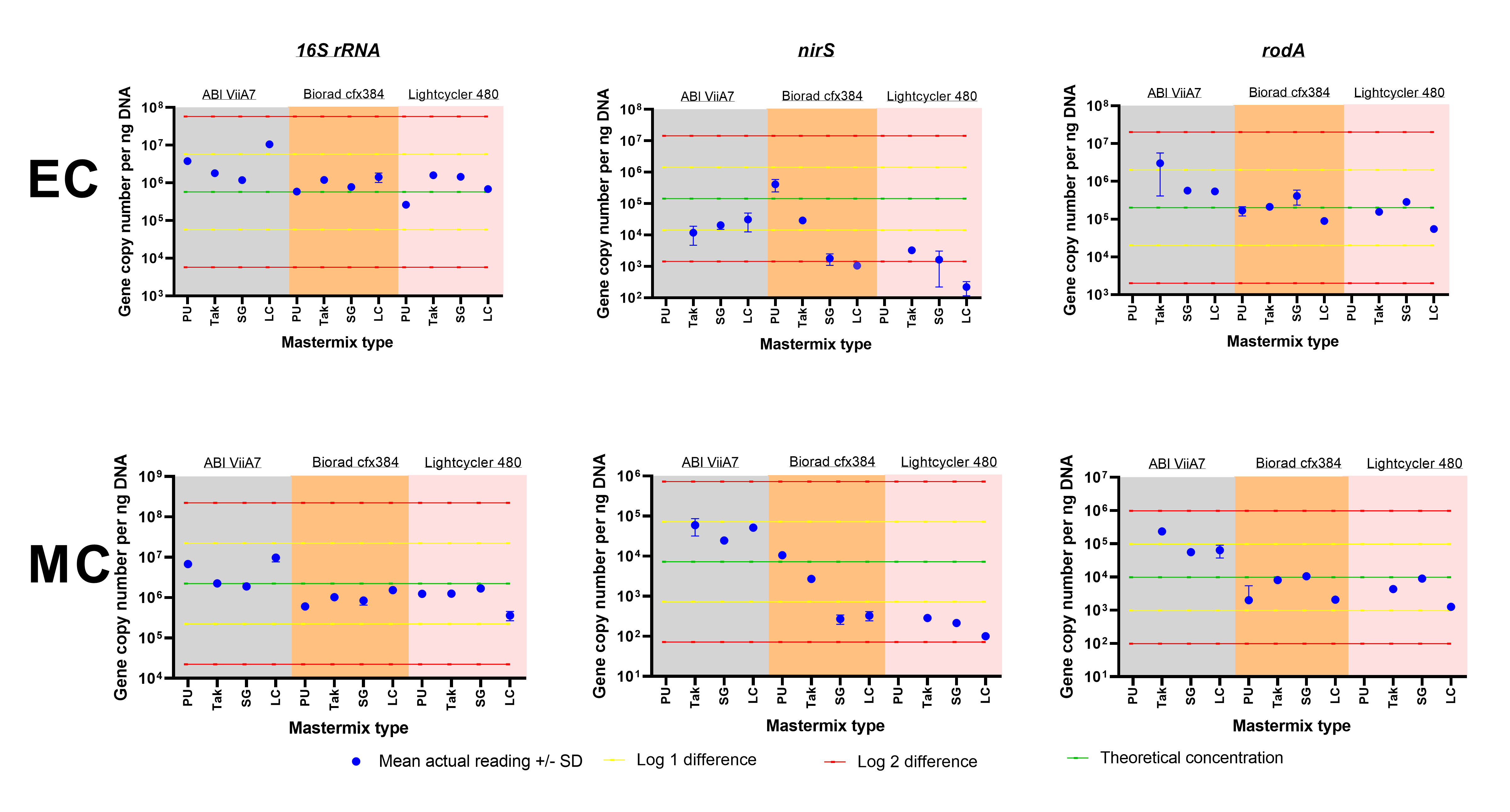

Supplement: fiaf073_Supplemental_Files [file fiaf073_supplemental_files.zip › Fig. S2 Supplementary Data.png]
